# A H_2_O_2_ self-sufficient nanoplatform with domino effects for thermal-responsive enhanced chemodynamic therapy†

**DOI:** 10.1039/c9sc05506a

**Published:** 2020-01-08

**Authors:** Shichao Zhang, Changyu Cao, Xinyi Lv, Hanming Dai, Zhihao Zhong, Chen Liang, Wenjun Wang, Wei Huang, Xuejiao Song, Xiaochen Dong

**Affiliations:** Key Laboratory of Flexible Electronics (KLOFE), Institute of Advanced Materials (IAM), School of Physical and Mathematical Sciences, Nanjing Tech University (NanjingTech) Nanjing 211800 China iamxcdong@njtech.edu.cn xjsong@njtech.edu.cn; School of Physical Science and Information Technology, Liaocheng University Liaocheng 252059 China; School of Chemistry and Materials Science, Nanjing University of Information Science & Technology Nanjing 210044 China; Shaanxi Institute of Flexible Electronics (SIFE), Northwestern Polytechnical University (NPU) Xi'an 710072 China

## Abstract

Chemodynamic therapy (CDT), employing Fenton or Fenton-like catalysts to convert hydrogen peroxide (H_2_O_2_) into toxic hydroxyl radicals (˙OH) to kill cancer cells, holds high promise in tumor therapy due to its high selectivity. However, the anticancer efficacy is unsatisfactory owing to the limited concentration of endogenous H_2_O_2_. Herein, thermal responsive nanoparticles with H_2_O_2_ self-sufficiency are fabricated by utilizing organic phase change materials (PCMs) to encapsulate iron–gallic acid nanoparticles (Fe–GA) and ultra-small CaO_2_. PCMs, acting as the gatekeeper, could be melted down by the hyperthermia effect of Fe–GA under laser irradiation with a burst release of Fe–GA and CaO_2_. The acidic tumor microenvironment would further trigger CaO_2_ to generate a large amount of H_2_O_2_ and Ca^2+^. The self-supplied H_2_O_2_ would be converted into ˙OH by participating in the Fenton reaction with Fe–GA. Meanwhile, *in situ* generation of Ca^2+^ could cause mitochondrial damage and lead to apoptosis of tumor cells. With efficient tumor accumulation illustrated in *in vivo* photoacoustic imaging, Fe–GA/CaO_2_@PCM demonstrated a superior *in vivo* tumor-suppressive effect without inducing systemic toxicity. The study presents a unique domino effect approach of PCM based nanoparticles with thermal responsiveness, H_2_O_2_ self-supply, and greatly enhanced CDT effects, showing bright prospects for highly efficient tumor treatment.

## Abstract 2

Phase change material based H_2_O_2_ self-supply nanoparticles for enhanced thermal responsive chemodynamic tumor therapy.

## Graphical abstract

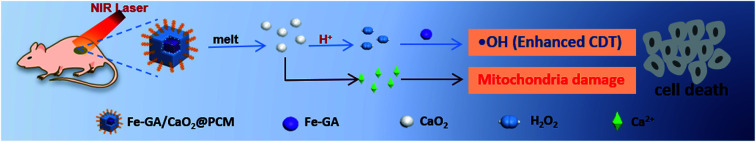

## Introduction

Chemodynamic therapy (CDT), a new type of reactive oxygen species (ROS)-based cancer treatment, depends on the *in situ* Fenton (Fenton-like) reaction to convert hydrogen peroxide (H_2_O_2_) into hydroxyl radicals (˙OH) by catalysis, and has high toxicity to cancer cells.^1–4^ Recently, various kinds of catalysts have been developed for CDT, such as Fe^2+^, Fe^3+^, Mn^2+^, Cu^+^, V^2+^ and Cr^4+^.^5–16^ Among these ions, Fe^2+^ and Fe^3+^ are typical Fenton ions and show great advantages in biocompatibility since iron is essential for cell growth, proliferation and oxygen delivery, as well as many other life processes. The typical Fenton reaction equation of iron is as follows:

1 Fe^2+^ + H_2_O_2_ → Fe^3+^ + OH^−^ + ˙OH

2
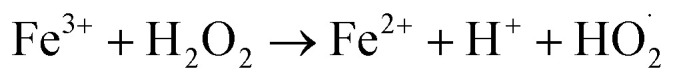


During such a cyclic process, no exogenous stimulation or oxygen is required. Compared with photodynamic therapy (PDT), the other kind of important ROS dependent therapy involving external laser irradiation and sufficient oxygen, CDT exhibits remarkable advantages in overcoming the obstacles of limited penetration depth of laser and hypoxic tumor microenvironments (TMEs).^17–20^ However, the chemodynamic efficacy is still restricted because of the limited endogenously produced H_2_O_2_, even though the concentration of H_2_O_2_ in many types of solid tumors has been reported to be higher than that in normal tissues.^8,20–22^ Under this circumstance, increasing the concentration of H_2_O_2_ or improving the catalytic capability of Fenton agents shows great promise in enhancing anticancer efficiency.

In response to the lack of H_2_O_2_ in tumors, various methods have been developed to increase the intratumoral H_2_O_2_ concentration.^8,15,20–22^ On the one hand, glucose oxidase loaded nanoparticles are utilized to oxidize intratumoral glucose to H_2_O_2_ and gluconic acid in the presence of O_2_*via* a glucose-metabolic reaction, increasing the H_2_O_2_ concentration. However, due to its oxygen dependence, the efficiency of H_2_O_2_ generation in this strategy is severely restricted by tumor hypoxia. On the other hand, metal peroxides (MPs), such as CuO_2_, have been utilized as H_2_O_2_ sources to produce H_2_O_2_ in acidic TMEs.^8,21^ Very recently, copper peroxide (CP) nanodots were successfully fabricated by Chen's group and used as an nanoagent to enhance CDT with self-supplying H_2_O_2_ in the acidic environment of endo/lysosomes.^8^ Although MPs show high efficiency in H_2_O_2_ generation, the poor stability in aqueous solution restricts their further application in cancer treatment, especially *via* intravenous injection. Therefore, developing a safer and more efficient nanocarrier, in which H_2_O_2_ production can be strictly controlled, is highly desirable for improving CDT efficiency.^23–26^

Organic phase-change materials (PCMs), which usually refer to materials with huge latent heats of fusion and exhibiting reversible solid–liquid transition at a nearly constant temperature, have attracted considerable interest in serving as thermo-responsive materials for drug release.^27–32^ Through adjusting the ratio of fatty acids or fatty alcohols, PCMs with different melting points (MPs) can be obtained. When the temperature is lower than the MP, the PCM plays the part of ‘gatekeeper’ to prevent the encapsulated drugs from premature release. Once the temperature is higher than the MP, a burst release of drugs can be achieved. In addition to small drugs, such as DOX, other kinds of materials, such as hydrophilic or hydrophobic nanoparticles, can also be encapsulated within PCMs, making them a promising candidate in the stimuli-sensitive drug delivery system.^33^

**Scheme 1:**
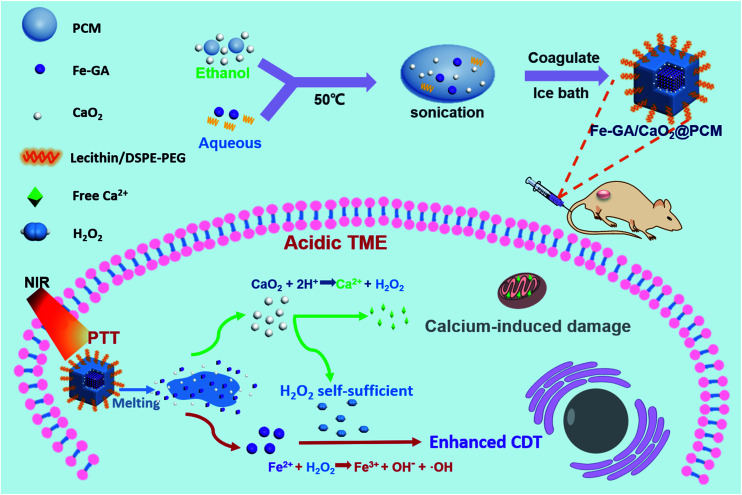
A scheme showing the fabrication of Fe–GA/CaO_2_@PCM and its application in H_2_O_2_ self-sufficient chemodynamic therapy.

Herein, organic PCMs with a melting point of 46 °C were utilized to co-encapsulate hydrophilic iron–gallic acid (Fe–GA) nanoparticles (NPs) and ultra-small hydrophobic CaO_2_ nanoparticles to obtain Fe–GA/CaO_2_@PCM NPs for thermal responsive enhanced CDT (Scheme 1). Fe–GA nanoparticles in Fe–GA/CaO_2_@PCM were used not only as a photothermal therapy (PTT) agent to generate the hyperthermia effect, but also an ideal CDT agent.^34–37^ The PCM layer could be the gatekeeper to isolate CaO_2_ from the outside environment, thus enhancing the stability and reducing the premature release of CaO_2_. When the Fe–GA/CaO_2_@PCM NPs were irradiated with an 808 nm laser, the hyperthermia induced by Fe–GA caused the melting of the PCM, causing the burst release of CaO_2_ NPs. The CaO_2_ NPs could further be triggered by acidic TMEs to produce a large amount of H_2_O_2_ and Ca^2+^, achieving high efficacy in Fe–GA based CDT and Ca^2+^ induced mitochondrial damage, respectively.^21,38^ Meanwhile, the PTT effect of Fe–GA could not only kill cancer cells, but also accelerate the generation of ˙OH.^3^ Under the guidance of photoacoustic imaging and fluorescence imaging, Fe–GA/CaO_2_@PCM NPs exhibited high accumulation in the tumor site *via* enhanced permeability and retention (EPR) effects. With the H_2_O_2_ self-sufficient CaO_2_ NPs, great performance in inhibiting tumor growth was shown in PTT/CDT combined treatment. Benefiting from their good biocompatibility and thermal responsiveness, the Fe–GA/CaO_2_@PCM NPs could be a potential multifunctional nanoplatform with on-demand H_2_O_2_ self-supply for enhanced PTT/CDT treatments.

## Results and discussion

**Fig. 1:**
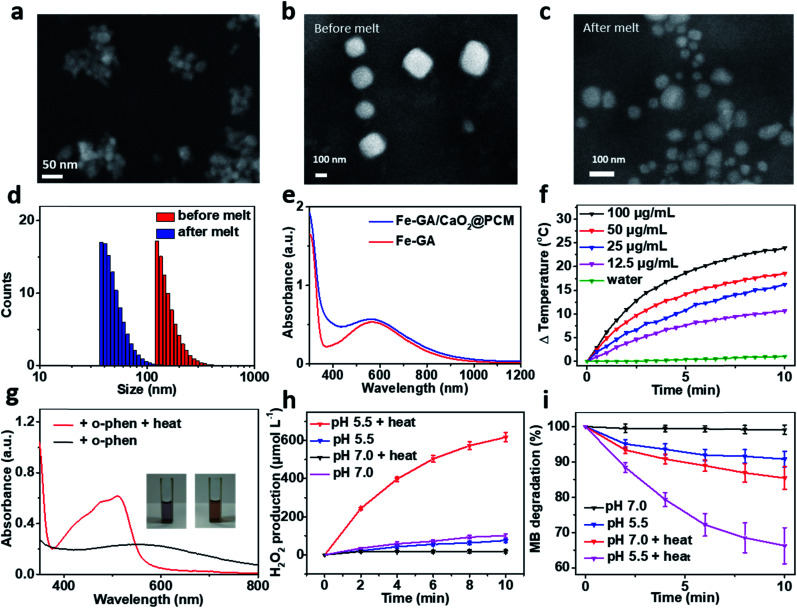
(a) SEM image of CaO_2_ NPs. SEM image of Fe–GA/CaO_2_@PCM NPs (b) before and (c) after melting. (d) Size distribution of Fe–GA/CaO_2_@PCM NPs before and after melting. (e) UV-vis absorption spectra of Fe–GA/CaO_2_@PCM NPs and Fe–GA. (f) Photothermal heating curve of different concentrations of Fe–GA/CaO_2_@PCM NPs (0–100 μg mL^−1^). (g) UV-vis absorption spectra of Fe–GA/CaO_2_@PCM NPs added with *o*-phen to detect Fe^2+^. (h) H_2_O_2_ generation from CaO_2_@PCM (100 μg mL^−1^) under different conditions. (i) MB degradation caused by Fe–GA/CaO_2_@PCM NP Fenton reaction under different conditions.

The thermal responsive Fe–GA/CaO_2_@PCM NPs were prepared as shown in Scheme 1. First, organic PCMs with a melting point of 46 °C were fabricated by mixing 1-hexadecanol with oleic acid at a ratio of 3.5 : 1 in ethanol. The solid PCM can be obtained for the following experiment *via* evaporating the ethanol. Next, Fe–GA NPs with an average size of 11 nm (Fig. S1a and b†) were successfully synthesized through a coordination method and ultra-small CaO_2_ NPs were synthesized according to the literature, respectively.^35,39^ The scanning electron microscopy (SEM) image and dynamic light scattering (DLS) revealed that the CaO_2_ NPs have a uniform size of about 18 nm (Fig. 1a and S1c†). X-ray diffraction (XRD) has confirmed the successful synthesis of CaO_2_ (Fig. S2a†). To obtain the Fe–GA/CaO_2_@PCM NPs, PCMs were utilized to co-encapsulate Fe–GA NPs and CaO_2_ NPs *via* a resolidification method. With the modification of lecithin and DSPE-mPEG, the Fe–GA/CaO_2_@PCM NPs exhibited excellent dispersibility and stability. The final capacity of CaO_2_ and Fe in Fe–GA/CaO_2_@PCM NPs was determined to be 8.1% and 12.7% (w/w) characterized by inductively coupled plasma mass spectrometry (ICP-MS), respectively. As revealed by the SEM and TEM images in Fig. 1b, c and S2b,† Fe–GA/CaO_2_@PCM NPs exhibit a cubic-like morphology with uniform distribution. Interestingly, upon laser irradiation, the NPs were melted down and the morphology turned out to be chaotic. Meanwhile, the diameters of the Fe–GA/CaO_2_@PCM NPs were decreased from 130 nm to 55 nm with laser irradiation (Fig. 1d). The sharply decreased diameter further confirms the thermal responsive profile of Fe–GA/CaO_2_@PCM NPs.

Owing to the encapsulation of Fe–GA NPs, Fe–GA/CaO_2_@PCM NPs exhibited high absorbance in the near infrared region (NIR) (Fig. 1e). The photothermal performance of Fe–GA/CaO_2_@PCM NPs was measured at different concentrations with 808 nm laser irradiation. Compared with water, the temperature of Fe–GA/CaO_2_@PCM NPs increased rapidly even at a low concentration (Fig. 1f). Meanwhile, Fe–GA/CaO_2_@PCM NPs still maintained good photothermal performance even after cycles of photothermal heating and cooling, presenting great photostability (Fig. S3a†). Calculated using the equation in Fig. S3b,† the photothermal conversion efficiency (*η*) of Fe–GA/CaO_2_@PCM NPs (100 μg mL^−1^) is as high as 49.8%. Moreover, the photothermal effects of Fe–GA@PCM NPs, CaO_2_@PCM NPs and Fe–GA/CaO_2_@PCM NPs irradiated with a constant power density (1.0 W cm^−2^, 10 min) were compared. As shown in Fig. S3c,† there were no significant differences between Fe–GA@PCM NPs and Fe–GA/CaO_2_@PCM NPs, while the temperature increase of CaO_2_@PCM NPs was similar to that of water, demonstrating that the PTT effect was contributed by Fe–GA nanoparticles.

Benefiting from PCM protection, Fe–GA/CaO_2_@PCM NPs exhibit excellent thermal responsiveness. To detect the release of Fe–GA, *o*-phenanthroline (*o*-phen) is utilized as an indicator, since it can form an orange-red complex with Fe^2+^ at pH 2–9. As shown in Fig. 1g, when the Fe–GA/CaO_2_@PCM NPs were placed at room temperature (25 °C), there was no significant change in the colour of the solution. Interestingly, a rapid change in the colour could be observed when the Fe–GA/CaO_2_@PCM NPs were under irradiation because of the melting of the PCM. The typical absorbance at 500 nm further demonstrated the thermal responsive release of Fe–GA. Next, the thermal responsive release of CaO_2_ and H_2_O_2_ generation under different conditions was investigated. Considering the reaction between Fe–GA and H_2_O_2_, CaO_2_@PCM NPs were chosen for the following experiment. KI, which could react with H_2_O_2_ to generate I^3−^, was used as the probe to detect H_2_O_2_ produced by CaO_2_ under different pH values by UV-vis measurements (Fig. S3d†). As shown in Fig. 1h, when the temperature of CaO_2_@PCM solution was heated to about 50 °C, the amount of produced H_2_O_2_ was calculated to be as high as 617 μmol L^−1^ under acidic conditions, which is far more than that in the solid tumors (less than 100 μmol L^−1^). However, no significant generation of H_2_O_2_ was found neither in the group of CaO_2_@PCM under neutral conditions with or without heating nor in the group of CaO_2_@PCM under acidic conditions without heating.

The above results demonstrated that the H_2_O_2_ self-supply behavior of CaO_2_@PCM could only be observed under acidic conditions with heating. Based on this, the Fenton effect of Fe–GA/CaO_2_@PCM NPs under different conditions was studied. The generation of ˙OH was demonstrated by electron paramagnetic resonance (EPR, Fig. S3e†) and a typical 1 : 2 : 2 : 1 signal could be observed in the EPR spectrum. Meanwhile, methylene blue (MB) was also used to detect the generation of ˙OH, for the absorption of MB at 660 nm decreases by reacting with ˙OH. With laser irradiation, the absorbance of MB decreased rapidly under acidic conditions, compared with the other groups (Fig. 1i), which is consistent with the results of H_2_O_2_ generation. Furthermore, *in vitro* thermal response release of Ca^2+^ was investigated by ICP-MS. A burst release of Ca^2+^ could be realized under 50 °C (Fig. S3f†).

**Fig. 2:**
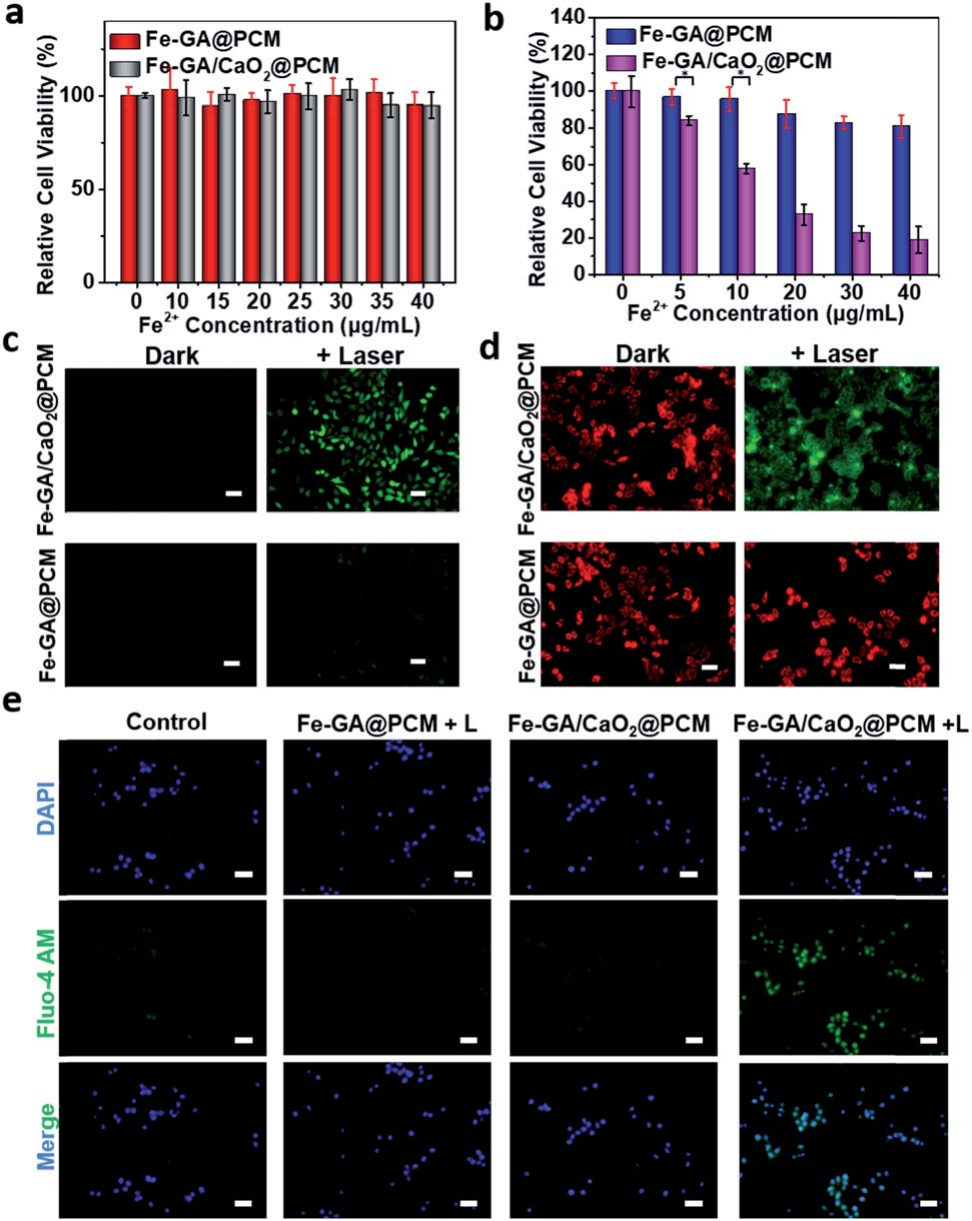
(a) Dark cytotoxicity of Fe–GA/CaO_2_@PCM NPs and Fe–GA@PCM NPs on HeLa cells. (b) Cytotoxicity of HeLa cells of Fe–GA/CaO_2_@PCM NPs and Fe–GA@PCM NPs at different concentrations under laser irradiation. *P < 0.001. (c) Intracellular hydroxyl radical detection with the DCFH-DA probe. (d) Mitochondrial membrane potential staining with the JC-1 probe. (e) Intracellular Ca^2+^ detection with the Fluo-4 AM probe. Scale bars: 100 μm.

The *in vitro* combined therapeutic efficiency of Fe–GA/CaO_2_@PCM NPs was evaluated. First, HeLa cells were incubated with CaO_2_@PCM NPs, Fe–GA@PCM NPs and Fe–GA/CaO_2_@PCM NPs at different concentrations for 24 h. There was no significant decrease in cell viability even at a high concentration (Fig. 2a and S4a†), indicating the great biocompatibility of the nanoparticles and the Wax-Sealed function of the PCM. Remarkably, when exposed to 808 nm laser irradiation, the cell viability in the group of Fe–GA/CaO_2_@PCM NPs was much lower than that in the group of Fe–GA@PCM NPs (Fig. 2b) with the same concentration of Fe. The results might be caused by the melting of the PCM under laser irradiation which triggered the release of CaO_2_, thus producing a large amount of H_2_O_2_, which could further react with released Fe ions to generate toxic ˙OH. The propidium iodide (PI) and calcein AM staining assay in Fig. S4b† has further demonstrated the cytotoxicity of Fe–GA/CaO_2_@PCM NPs.

To study the intracellular action mechanism of Fe–GA/CaO_2_@PCM NPs as the H_2_O_2_ self-supplier to kill cancer cells, 2,7-dichlorofluorescein diacetate (DCFH-DA) staining assay was utilized to confirm the generation of hydroxyl radicals (Fig. 2c). Without laser irradiation, HeLa cells in the groups of Fe–GA/CaO_2_@PCM and Fe–GA@PCM exhibited weak green fluorescence. When the cells were exposed to laser irradiation, no obvious green fluorescence could be observed in Fe–GA@PCM, which might be owing to the limited content of intracellular H_2_O_2_. In contrast, strong green fluorescence was observed in cells treated with an Fe–GA/CaO_2_@PCM + laser, indicating the generation of a large amount of hydroxyl radicals in cells. According to the reaction of CaO_2_ under acidic conditions, not only H_2_O_2_ but also Ca^2+^ could be burst generated upon laser irradiation. The excessive Ca^2+^ in the cells will damage intracellular proteins and nucleic acids, as well as organelles especially mitochondria, leading to cell death. Therefore, the intracellular generation of Ca^2+^ was further investigated. Consistent with the results in ROS detection, strong green fluorescence can be detected only in the group of Fe–GA/CaO_2_@PCM + laser, demonstrating the burst release of Ca^2+^ (Fig. 2e). The mitochondrion, acting as an indispensable organelle in cell energy conversion and apoptosis, is vulnerable to excessive Ca^2+^ and reactive oxygen species. To characterize mitochondrial damage, the JC-1 fluorescence probe was employed to detect the mitochondrial membrane potential (MMP) of cells. Compared with the groups of the Fe–GA/CaO_2_@PCM or Fe–GA@PCM without irradiation and Fe–GA@PCM with irradiation, fluorescence in the Fe–GA/CaO_2_@PCM + laser turned from red to green (Fig. 2d), indicating that MMP declined and cellular apoptosis occurred under such a therapy. These results demonstrate that Fe–GA/CaO_2_@PCM acts as an efficient H_2_O_2_ self-supplier with great potential in combined PTT/CDT.

**Fig. 3:**
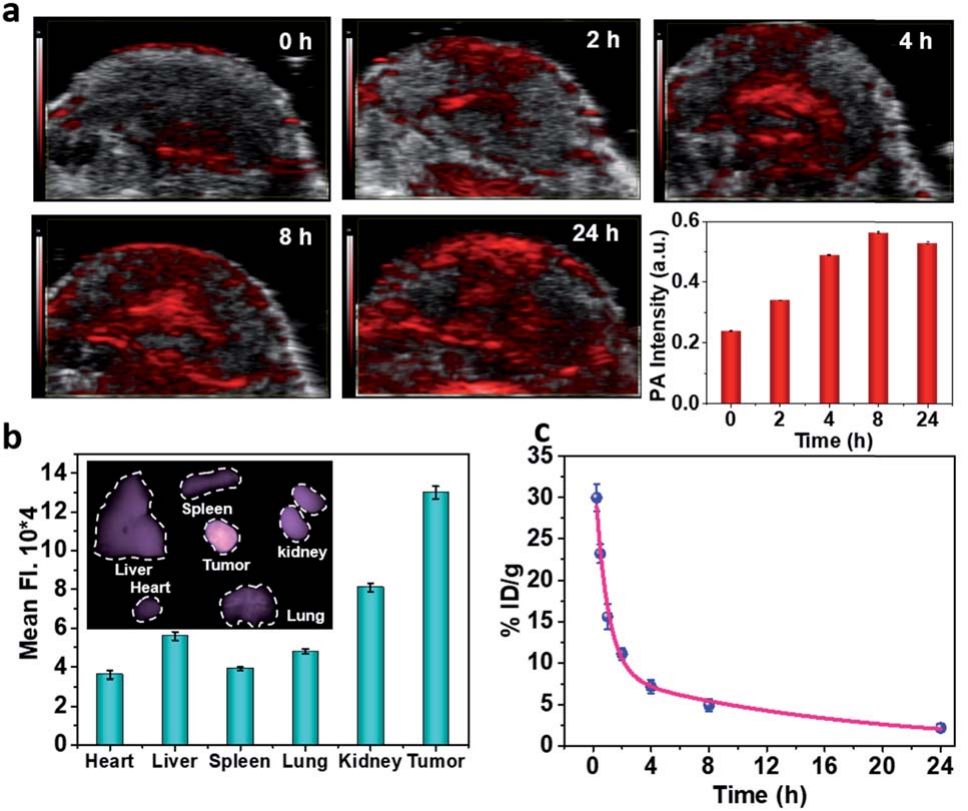
(a) *In vivo* PA images of HeLa xenograft mice and quantification of the PA signal several time points after i.v. injection with Fe–GA/CaO_2_@PCM NPs. (b) *Ex vivo* fluorescence images of major organs and tumors 24 h after injection and quantification of the fluorescence signal. (c) Concentration–time curve of Fe–GA/CaO_2_@PCM NPs in mice after i.v. injection. The concentration refers to the Fe ion content detected by ICP-MS.

To realize more accurate therapy and reduce damage on normal tissues, imaging guidance is beneficial to trace the nanoparticles and provide the information on tumors. Herein, taking advantage of the absorbance of Fe–GA in the NIR, photoacoustic (PA) imaging was conducted after intravenously injected with Fe–GA/CaO_2_@PCM NPs. PA signals in the tumor site appeared at 2 h and gradually enhanced with time (Fig. 3a). Meanwhile, IR780 (a commonly used fluorescent dye) was encapsulated within Fe–GA/CaO_2_@PCM NPs to explore the biodistribution of Fe–GA/CaO_2_@PCM NPs (Fig. 3b). 24 h after intravenous injection of Fe–GA/CaO_2_@PCM NPs, the main organs and tumors of HeLa tumor-bearing mice were obtained for *ex vivo* fluorescence imaging. A strong fluorescence signal could be observed in the tumor site, confirming the high tumor uptake *via* the EPR effect. Meanwhile, the blood circulation study was carefully carried out by detecting the concentration of the Fe ion content in blood samples by ICP-MS. The obtained data were fitted to a time-dependent concentration curve using a two-compartment model, in which the diffusion half-time and elimination half-time were calculated to be 0.6 ± 0.14 h and 11.55 ± 1.43 h, respectively (Fig. 3c). Benefiting from the high tumor accumulation and long blood circulation, Fe–GA/CaO_2_@PCM NPs show great potential for application in cancer therapy.

**Fig. 4:**
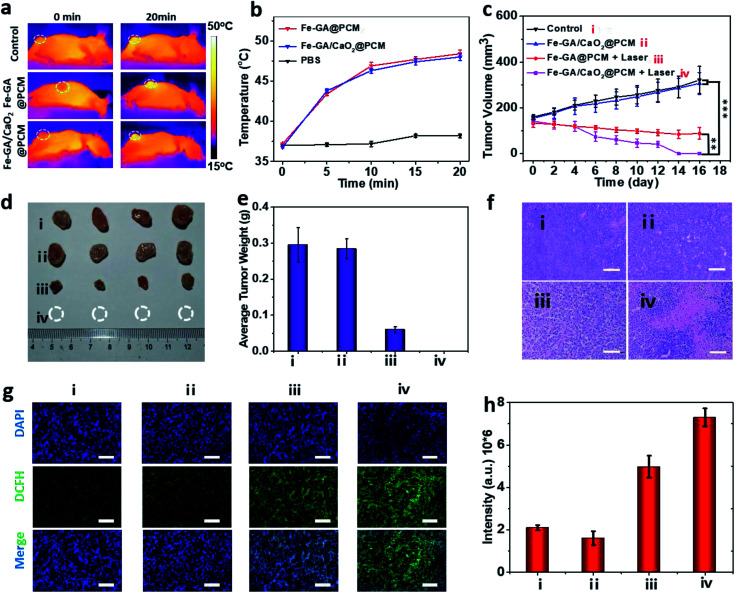
(a) IR thermal images of mice bearing HeLa tumors irradiated with an 808 nm laser (1.0 W cm^−2^, 20 min). (b) The temperature changes in the tumor site under laser irradiation. (c) Tumor volume curves of each group: **P < 0.01 and ***P < 0.01. (d) Resected tumors of each group on day 16. (e) Average tumor weight of mice in each group after 16 days. (f) H&E stained images of tumor sections from each treatment group. Scale bars: 100 μm. (g) Immunofluorescence staining of DCFH-DA to detect intratumoral hydroxyl radicals after different treatments. Scale bars: 50 μm. (h) Semiquantitative analysis of fluorescence signals with DCFH-DA immunofluorescence staining.

To evaluate PTT & CDT synergistic treatment effects of Fe–GA/CaO_2_@PCM NPs *in vivo*, nude mice bearing HeLa tumors were divided into 4 groups randomly: (i) i.v. injected with saline (200 μL). (ii) i.v. injected with Fe–GA/CaO_2_@PCM NPs (200 μL, 1.5 mg kg^−1^) and without laser irradiation. (iii) i.v. injected with Fe–GA@PCM NPs (200 μL, 1.5 mg kg^−1^) and irradiated with an 808 nm laser. (iv) i.v. injected with Fe–GA/CaO_2_@PCM NPs (200 μL, 1.5 mg kg^−1^) and irradiated with an 808 nm laser. All the illumination was given 6 h after injection with a constant power density (1.0 W cm^−2^, 20 min). The temperature changes in the tumor site under laser irradiation were detected with an infrared camera (Fig. 4a and b). The temperature in groups of Fe–GA/CaO_2_@PCM NPs and Fe–GA@PCM NPs rapidly increased to about 47.5 °C, which was sufficient for the melting of the PCM to trigger the release of CaO_2_. During the treatment period, the tumor volume change of each group was recorded with a digital caliper every two days (Fig. 4c). No noticeable tumor growth inhibition effect was observed in group ii, which might due to the perfect protection of PCM. When laser irradiation was added, the hyperthermia effect generated by Fe–GA@PCM NPs induced a partial anticancer effect. Remarkably, owing to the self-sufficient H_2_O_2_ enhanced CDT by thermal responsive Fe–GA/CaO_2_@PCM, tumor growth in the mice treated with Fe–GA/CaO_2_@PCM NPs plus laser irradiation was completely ablated without recurrence in the period of therapy (Fig. 4d, e and S5a†). The high efficacy of combined PTT/CDT was also confirmed using the haematoxylin and eosin (H&E) staining tumor slices after different treatments (Fig. 4f). Consistent with the tumor growth, compared with the other three groups, the most severe damage was observed in the tumors treated with Fe–GA/CaO_2_@PCM NPs plus laser irradiation. To further study the ability of Fe–GA/CaO_2_@PCM NPs to self-supply H_2_O_2_ under laser irradiation, an *ex vivo* DCFH-DA staining assay was conducted to confirm ˙OH generation. Weak green fluorescence could be detected in the control group or tumor treated with Fe–GA/CaO_2_@PCM NPs. A slightly enhanced fluorescence signal was observed in tumors treated with Fe–GA@PCM NPs plus laser irradiation, which might be owing to the released Fe–GA reacted with the endogenous H_2_O_2_ to generate ˙OH. Significantly, strong green fluorescence appeared in the tumors treated with Fe–GA/CaO_2_@PCM NPs plus laser irradiation owing to the on-demand generation of a large amount of H_2_O_2_ (Fig. 4g and h). The results demonstrate that Fe–GA/CaO_2_@PCM NPs could serve as a H_2_O_2_ self-sufficient nanoplatform for enhanced PTT/CDT. Meanwhile, there was no significant decrease in the mouse body weights during the therapeutic period (16 days), suggesting no acute toxicity of the Fe–GA/CaO_2_@PCM NPs (Fig. S5b†). H&E staining slices of major organs and tissues revealed no obvious damage, further indicating the biosafety of Fe–GA/CaO_2_@PCM NPs (Fig. S6†).

## Conclusions

In summary, we developed an intelligent nanoplatform by co-encapsulating Fe–GA and CaO_2_ in organic PCMs, and their application as a thermal responsive CDT agent with self-supplied H_2_O_2_. PCMs, as the protective layer, could be melted by the hyperthermia induced by Fe–GA under laser irradiation, thus releasing Fe–GA and CaO_2_. In acidic TMEs, CaO_2_ could serve as a H_2_O_2_ supplier to generate a large amount of H_2_O_2_ and Ca^2+^ as well. The overproduced H_2_O_2_ could be converted into toxic ˙OH by Fe–GA, achieving an enhanced CDT effect. Meanwhile, the burst release of Ca^2+^ in cancer cells further caused damage to mitochondria and led to cell apoptosis. Owing to the modification of polyethylene glycol, the Fe–GA/CaO_2_@PCM NPs exhibited a long blood circulation and high tumor uptake. With the imaging-guidance, *in vivo* combined PTT/CDT was carried out and the interlock performance of Fe–GA/CaO_2_@PCM NPs enabled a superior tumor-suppressive effect under laser irradiation without inducing noticeable damage to normal tissues or organs. Compared with other methods to improve the intratumoral concentration of H_2_O_2_, the work presented an intelligent and much safer nanoplatform with thermal and acidic TME responsiveness. It is worthy to expect that the development of smart types of multifunctional agents with high-performance may provide new possibilities for the potential clinical translation of PTT/CDT.

## Experimental section

### Synthesis of Fe–GA nanoparticles

100 mg of polyvinylpyrrolidone (PVP) was added into 10 mL DI water under sonication at 25 °C. Then, 0.1 mL of FeCl_2_ aqueous solution (200 mg mL^−1^) was mixed with the PVP solution under vigorous stirring. 20 mg of GA was dispersed in 1 mL of water for use. After 30 min of stirring, the FeCl_2_–PVP mixture was mixed with GA solution and stirred overnight. After being filtered with the PP microporous membrane (0.22 μm), the resultant product was stored in a 4 °C refrigerator for the following use.

### Synthesis of CaO_2_ nanoparticles

45 mL of PEG-200 and 1 mL of CaCl_2_ aqueous solution (0.2 g mL^−1^) were added into a round bottom flask and stirred for 15 minutes. After adding 0.55 mL of ammonia solution (1.0 M), the mixture was stirred for 10 minutes. Then, 1 mL 30% H_2_O_2_ solution was slowly added into the mixture using a syringe. After stirring for 6 hours, a clear transparent liquid was obtained. After slowly adding 4 mL of NaOH solution (1.0 mol L^−1^) with stirring, the liquid turned into white turbid liquid. The final products were obtained by centrifugation with DI water and ethanol. CaO_2_ nanoparticles were stored in ethanol at 4 °C.

### Preparation of PCMs

PCMs were synthesized according to previous studies.^29^ In brief, 40 mg of oleic acid and 140 mg of 1-hexadecanol were added into 10 mL absolute ethanol. After sonication, PCMs were obtained and stored at 4 °C for use.

### Preparation of Fe–GA/CaO_2_@PCM nanoparticles

Fe–GA/CaO_2_@PCM NPs were formed by a resolidification method. In brief, 0.4 mL CaO_2_ ethanol solution (2 mg mL^−1^) and 0.4 mL of PCM ethanol solution (18 mg mL^−1^) were mixed and used as solution 1.6 mg DSPE-mPEG-MW2000 and 20 mg of l-α-lecithin were added into 10 mL of Fe–GA aqueous solution (1 mg mL^−1^) and used as solution 2. Under sonication at 50 °C, solution 1 was added into solution 2 and then rapidly cooled in an ice bath. The purple dispersion of Fe–GA/CaO_2_@PCM NPs was obtained after dialyzing against DI water for 24 h. The concentration of iron and calcium was determined by inductively coupled plasma mass spectrometry (ICP-MS).

### *In vitro* H_2_O_2_ production

H_2_O_2_ can react with KI to generate I^3−^ which resulted in an absorption peak at 350 nm; the standard curve can be used to quantitatively analyze the hydrogen peroxide concentration in the solution. The CaO_2_@PCM NPs were divided into three groups (*n* = 3): (i) CaO_2_@PCM NPs dispersed in DI water (pH 7.0, 100 μg mL^−1^) at 25 °C. (ii) CaO_2_@PCM NPs dispersed in PBS buffer (pH 5.5, 100 μg mL^−1^) at 25 °C. (iii) CaO_2_@PCM NPs dispersed in PBS buffer (pH 5.5, 100 μg mL^−1^) at 50 °C. 20 μL of KI (0.1 M) aqueous solution was added to each group. The UV-vis absorption spectra of solution were measured every 2 minutes to calculate the concentration of generated H_2_O_2_.

### Fenton effect

Methylene blue was used to detect the hydroxyl radicals generated by the Fenton reaction. The Fe–GA/CaO_2_@PCM NPs were divided into four groups (*n* = 3): (i) Fe–GA/CaO_2_@PCM NPs dispersed in DI water (pH 7.0, 100 μg mL^−1^) at 25 °C. (ii) Fe–GA/CaO_2_@PCM NPs dispersed in DI water (pH 7.0, 100 μg mL^−1^) at 50 °C. (iii) Fe–GA/CaO_2_@PCM NPs dispersed in PBS buffer (pH 5.5, 100 μg mL^−1^) at 25 °C. (iv) Fe–GA/CaO_2_@PCM NPs dispersed in PBS buffer (pH 5.5, 100 μg mL^−1^) at 50 °C. The decrease of absorption at 660 nm of each group was recorded every 2 minutes to quantitatively analyze the degradation of methylene blue, which represented the production of hydroxyl radicals.

### Photothermal effect

Photothermal heating curves under 808 nm laser irradiation (1.0 W cm^−2^, 10 min) of different nanoparticles (Fe–GA@PCM, Fe–GA/CaO_2_@PCM and CaO_2_@PCM) and different concentrations (100, 50, 25, 12.5 and 0 μg mL^−1^) of Fe–GA/CaO_2_@PCM NPs were measured by using an FLIR infrared camera. Furthermore, Fe–GA/CaO_2_@PCM NPs were tested under 5 photothermal cycles with 808 nm laser irradiation (1.0 W cm^−2^, 10 min) to validate the photostability.

### Thermal response release of Ca^2+^

To investigate the thermal responsive release of Ca^2+^, 3 mL of Fe–GA/CaO_2_@PCM (100 μg mL^−1^) was dialyzed at 50 °C and 25 °C in 30 mL H_2_O, respectively. The liquid outside the dialysis bag was collected every 5 min under continuous stirring. The Ca content was detected by ICP-MS.

### *In vitro* cytotoxicity

HeLa cells were seeded into 96-well plates and incubated for 24 h under standard conditions (37 °C and 5% CO_2_). The medium was replaced by different concentrations of Fe–GA/CaO_2_@PCM NP and Fe–GA@PCM NP medium solutions. For dark toxicity, the cells were incubated in the dark for 24 h, and for phototoxicity, the cells were irradiated with an 808 nm laser (3 min, 1.0 W cm^−2^) and were incubated for 12 h. 3-(4,5-Dimethyl-2-thiazolyl)-2,5-diphenyl-2-*H*-tetrazolium bromide (MTT) assays were conducted to measure the relative cell viability.

### Intracellular detection of ˙OH

A 2,7-dichlorodi-hydrofluorescein diacetate (DCFH-DA, Sigma-Aldrich) probe was used to detect the intracellular hydroxyl radicals produced by the Fenton effect according to the standard protocol. The HeLa cells were divided into four groups with different treatments: (i) added with 20 μg mL^−1^ of Fe–GA/CaO_2_@PCM NPs in the dark; (ii) added with 20 μg mL^−1^ of Fe–GA/CaO_2_@PCM NPs with 808 nm laser irradiation (1.0 W cm^−2^, 10 min); (iii) added with 20 μg mL^−1^ of Fe–GA@PCM NPs in the dark; (iv) added with 20 μg mL^−1^ of Fe–GA@PCM NPs with 808 nm laser irradiation (1.0 W cm^−2^, 10 min).

### Detection of mitochondrial membrane potential

The JC-1 fluorescent probe was employed to detect the changes of mitochondrial membrane potential. HeLa cells were seeded in a 6-well plate and incubated in the dark for 24 h under standard conditions (37 °C and 5% CO_2_). Fe–GA/CaO_2_@PCM NPs (20 μg mL^−1^) and Fe–GA@PCM NPs (20 μg mL^−1^) were added in the medium and incubated for 6 h, respectively. The illumination group was irradiated with an 808 nm laser at 1.0 W cm^−2^ for 10 min. 2 h after incubation, the cells were stained with JC-1 for 40 min. After washing with PBS buffer 3 times, the fluorescence of JC-1 was observed with an inverted fluorescence microscope.

### Intracellular detection of Ca^2+^

Fluo-4 AM is a commonly used fluorescent probe for detecting the intracellular concentration of Ca^2+^. HeLa cells were seeded in a 6-well plate. After being incubated at 37 °C for 24 h, the cells were stained with Fluo-4 AM and DAPI according to the standard method. The fluorescence was then detected using a fluorescence microscope.

### *In vivo* imaging

Photoacoustic (PA) imaging and *in vivo* fluorescence imaging were used to observe the enrichment of nanoparticles in the tumor site after intravenous injection. For PA imaging, after being i.v. injected with Fe–GA/CaO_2_@PCM NPs, the tumor regions of mice were imaged with a Visual sonics Vevo 2100 LAZR system at different time points (0, 2, 4, 8 and 24 h) with 808 nm laser excitation.

### Biodistribution and blood circulation study

To explore the biodistribution of Fe–GA/CaO_2_@PCM NPs, the fluorescent dye IR780 was added into Fe–GA/CaO_2_@PCM NPs to provide near-infrared fluorescence. After being intravenously injected with the above Fe–GA/CaO_2_@PCM NPs, the major organs and tumors of HeLa xenograft mice were taken out 24 h later, and the fluorescence intensity was detected with a Fluor Vivo 200 (INDEC Biosystems, USA) with 760 nm for excitation to analyze the biodistribution of the nanoparticles. To study the blood circulation of nanoparticles *in vivo*, mice were intravenously injected with Fe–GA/CaO_2_@PCM NPs (Fe injection dose: 1.5 mg kg^−1^), and blood samples were taken out at several time points (0, 0.25, 0.5, 1.0, 1.5, 3.0, 6.0, 9.0, and 24 h). The concentration of Fe in the blood was determined by ICP.

### *In vivo* tumor therapy

Nude mice bearing HeLa tumors were divided into 4 groups randomly (*n* = 4): (i) i.v. injected with saline (200 μL); (ii) i.v. injected with Fe–GA/CaO_2_@PCM NPs (200 μL, 1.5 mg kg^−1^) without irradiation; (iii) i.v. injected with Fe–GA@PCM NPs (200 μL, 1.5 mg kg^−1^) and irradiated with an 808 nm laser (1.0 W cm^−2^, 20 min); (iv) i.v. injected with Fe–GA/CaO_2_@PCM NPs (200 μL, 1.5 mg kg^−1^) and irradiated with an 808 nm laser (1.0 W cm^−2^, 20 min). The temperature changes in the tumor site were detected with an infrared camera. The tumor sizes and weight of mice were recorded every 2 days for 16 days. After treatment, tumors and other main organs of mice were taken out for hematoxylin and eosin (H&E) staining to study systemic toxicity.

### *In vivo* ROS staining

Tumor-bearing mice received the same treatment as the therapy group. Tumors were immediately taken out after treatment for cryosection. With DCFH-DA staining, the production of hydroxyl radicals in tumors was observed by fluorescence microscopy.

## Ethical statement

All animal experiments were performed according to the NIH guidelines for the care and use of laboratory animals. All the experiments were approved by the School of Pharmaceutical Science in Nanjing Tech University.

## Conflicts of interest

There are no conflicts of interest to declare.

## Supplementary Material

SC-011-C9SC05506A-s001

## References

[cit1] Tang Z., Liu Y., He M., Bu W. (2019). Angew. Chem..

[cit2] Lin H., Chen Y., Shi J. (2018). Chem. Soc. Rev..

[cit3] Ranji-Burachaloo H., Gurr P., Dunstan D., Qiao G. (2018). ACS Nano.

[cit4] Huo M., Wang L., Chen Y., Shi J. (2017). Nat. Commun..

[cit5] Bokare A., Choi W. (2014). J. Hazard. Mater..

[cit6] Dai Y., Yang Z., Cheng S., Wang Z., Zhang R., Zhu G., Wang Z., Yung B., Tian R., Jacobson O., Xu C., Ni Q., Song J., Sun X., Niu G., Chen X. (2018). Adv. Mater..

[cit7] Li T., Zhou J., Wang L., Zhang H., Song C., de la Fuente J., Pan Y., Song J., Zhang C., Cui D. (2019). Adv. Healthcare Mater..

[cit8] Lin L., Huang T., Song J., Ou X., Wang Z., Deng H., Tian R., Liu Y., Wang J., Liu Y., Yu G., Zhou Z., Wang S., Niu G., Yang H., Chen X. (2019). J. Am. Chem. Soc..

[cit9] Lin L., Song J., Song L., Ke K., Liu Y., Zhou Z., Shen Z., Li J., Yang Z., Tang W., Niu G., Yang H., Chen X. (2018). Angew. Chem..

[cit10] Liu Y., Zhen W., Jin L., Zhang S., Sun G., Zhang T., Xu X., Song S., Wang Y., Liu J., Zhang H. (2018). ACS Nano.

[cit11] Ma B., Wang S., Liu F., Zhang S., Duan J., Li Z., Kong Y., Sang Y., Liu H., Bu W., Li L. (2019). J. Am. Chem. Soc..

[cit12] Ma P., Xiao H., Yu C., Liu J., Cheng Z., Song H., Zhang X., Li C., Wang J., Gu Z., Lin J. (2017). Nano Lett..

[cit13] Valko M., Jomova K., Rhodes C., Kuca K., Musilek K. (2016). Arch. Toxicol..

[cit14] Zhang L., Wan S., Li C., Xu L., Cheng H., Zhang X. (2018). Nano Lett..

[cit15] Zhou Z., Song J., Tian R., Yang Z., Yu G., Lin L., Zhang G., Fan W., Zhang F., Niu G., Nie L., Chen X. (2017). Angew. Chem..

[cit16] Chen Q., Feng L., Liu J., Zhu W., Dong Z., Wu Y., Liu Z. (2016). Adv. Mater..

[cit17] Agostinis P., Berg K., Cengel K., Foster T., Girotti A., Gollnick S., Hahn S., Hamblin M., Juzeniene A., Kessel D., Korbelik M., Moan J., Mroz P., Nowis D., Piette J., Wilson B., Golab J. (2011). Ca-Cancer J. Clin..

[cit18] Estrella V., Chen T., Lloyd M., Wojtkowiak J., Cornnell H., Ibrahim-Hashim A., Bailey K., Balagurunathan Y., Rothberg J., Sloane B., Johnson J., Gatenby R., Gillies R. (2013). Cancer Res..

[cit19] Wang Z., Zhang Y., Ju E., Liu Z., Cao F., Chen Z., Ren J., Qu X. (2018). Nat. Commun..

[cit20] Ranji-Burachaloo H., Reyhani A., Gurr P., Dunstan D., Qiao G. (2019). Nanoscale.

[cit21] Zhang M., Song R., Liu Y., Yi Z., Meng X., Zhang J., Tang Z., Yao Z., Liu Y., Liu X., Bu W. (2019). Chem.

[cit22] Liu X., Liu Y., Wang J., Wei T., Dai Z. (2019). ACS Appl. Mater. Interfaces.

[cit23] Souho T., Lamboni L., Xiao L., Yang G. (2018). Biotechnol. Adv..

[cit24] Bertrand N., Wu J., Xu X., Kamaly N., Farokhzad O. (2014). Adv. Drug Delivery Rev..

[cit25] Biju V. (2014). Chem. Soc. Rev..

[cit26] Kelkar S., Reineke T. (2011). Bioconjug. Chem..

[cit27] Dai Y., Su J., Wu K., Ma W., Wang B., Li M., Sung P., Shen Q., Wang Q., Fan Q. (2019). ACS Appl. Mater. Interfaces.

[cit28] Li Q., Sun L., Hou M., Chen Q., Yang R., Zhang L., Xu Z., Kang Y., Xue P. (2019). ACS Appl. Mater. Interfaces.

[cit29] Liu G., Zhang S., Shi Y., Huang X., Tang Y., Chen P., Si W., Huang W., Dong X. (2018). Adv. Funct. Mater..

[cit30] Sun Q., He F., Bi H., Wang Z., Sun C., Li C., Xu J., Yang D., Wang X., Gai S., Yang P. (2019). Chem. Eng. J..

[cit31] Yuan Y., Zhang N., Tao W., Cao X., He Y. (2014). Renewable Sustainable Energy Rev..

[cit32] Yuan Z., Qu S., He Y., Xu Y., Liang L., Zhou X., Gu L., Gu Y., Chen H. (2018). Biomater. Sci..

[cit33] Li X., Kim J., Yoon J., Chen X. (2017). Adv. Mater..

[cit34] An L., Yan C., Mu X., Tao C., Tian Q., Lin J., Yang S. (2018). ACS Appl. Mater. Interfaces.

[cit35] Jin Q., Zhu W., Jiang D., Zhang R., Kutyreff C., Engle J., Huang P., Cai W., Liu Z., Chen L. (2017). Nanoscale.

[cit36] Wang Y., Zhang J., Zhang C., Li B., Wang J., Zhang X., Li D., Sun S. (2018). ACS Sustainable Chem. Eng..

[cit37] Zeng J., Cheng M., Wang Y., Wen L., Chen L., Li Z., Wu Y., Gao M., Chai Z. (2016). Adv. Healthcare Mater..

[cit38] Chen Q., Huo D., Cheng H., Lyu Z., Zhu C., Guan B., Xia Y. (2018). Adv. Healthcare Mater..

[cit39] Liu L., Zhang Y., Qiu W., Zhang L., Gao F., Li B., Xu L., Fan J., Li Z., Zhang X. (2017). Small.

