# Correction: A H_2_O_2_ self-sufficient nanoplatform with domino effects for thermal-responsive enhanced chemodynamic therapy

**DOI:** 10.1039/d3sc90165c

**Published:** 2023-08-25

**Authors:** Shichao Zhang, Changyu Cao, Xinyi Lv, Hanming Dai, Zhihao Zhong, Chen Liang, Wenjun Wang, Wei Huang, Xuejiao Song, Xiaochen Dong

**Affiliations:** a Key Laboratory of Flexible Electronics (KLOFE), Institute of Advanced Materials (IAM), School of Physical and Mathematical Sciences, Nanjing Tech University (NanjingTech) Nanjing 211800 China iamxcdong@njtech.edu.cn xjsong@njtech.edu.cn; b School of Physical Science and Information Technology, Liaocheng University Liaocheng 252059 China; c School of Chemistry and Materials Science, Nanjing University of Information Science & Technology Nanjing 210044 China; d Shaanxi Institute of Flexible Electronics (SIFE), Northwestern Polytechnical University (NPU) Xi’an 710072 China

## Abstract

Correction for ‘A H_2_O_2_ self-sufficient nanoplatform with domino effects for thermal-responsive enhanced chemodynamic therapy’ by Shichao Zhang *et al.*, *Chem. Sci.*, 2020, **11**, 1926–1934, https://doi.org/10.1039/C9SC05506A.

It has come to the authors’ attention that there was one error in Fig. S6 in the ESI. An incorrect image for the H&E stained liver for the Fe-GA/CaO_2_@PCM Dark group (row 2, column 2) was mistakenly used due to carelessness when editing the figure. The corrected version is displayed below. The data analysis and conclusions in the paper remain unchanged.


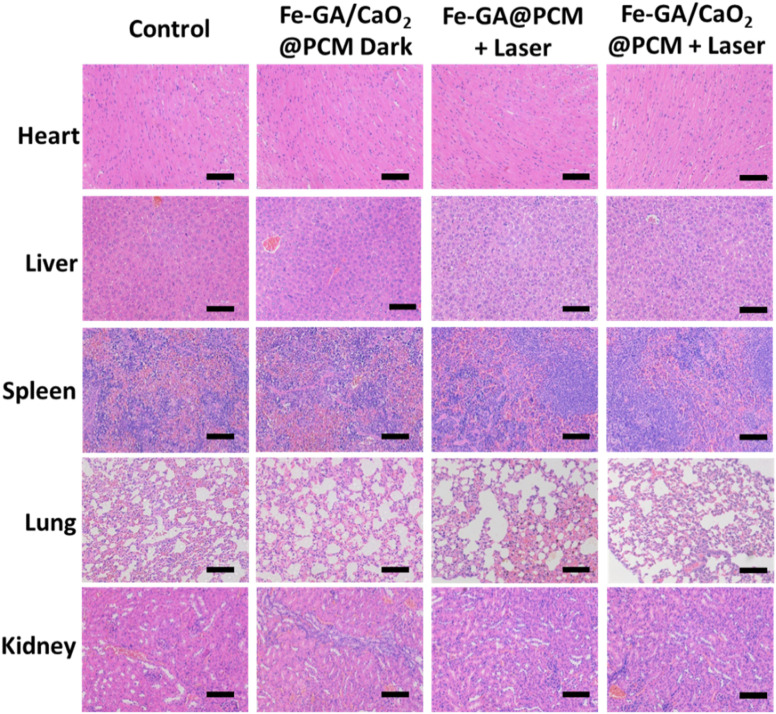
 **Fig. S6** H&E staining of organs from each group of mice after different treatment. Scale bar: 100 μm.

 

The Royal Society of Chemistry apologises for these errors and any consequent inconvenience to authors and readers.

## Supplementary Material

