# Supplementary material for: A H2O2 self-sufficient nanoplatform with domino effects for thermal-responsive enhanced chemodynamic therapy
Source: Chem Sci. 2020 Jan 8;11(7):1926–34. doi: 10.1039/c9sc05506a (PMC8148300; doi:10.1039/c9sc05506a)
Supplement: SC-011-C9SC05506A-s001 [file SC-011-C9SC05506A-s001.pdf]

Electronic Supplementary Material (ESI) for Chemical Science.
This journal is © The Royal Society of Chemistry 2020

H_2O_2 Self-Sufficient Nanoplatform with Domino-effect for Thermal-Responsive Enhanced Chemodynamic Therapy

Shichao Zhang[†], Changyu Cao[†], Xinyi Lv[†], Hanming Dai[†], Zhihao Zhong[†], Chen Liang[†], Wenjun Wang[‡], Wei Huang[±], Xuejiao Song[†], Xiaochen Dong[†§]**

[†]Key Laboratory of Flexible Electronics (KLOFE) and Institute of Advanced Materials (IAM), School of Physical and Mathematical Sciences, Nanjing Tech University (NanjingTech), Nanjing 211800, China

E-mail: iamxcdong@njtech.edu.cn; xjsong@njtech.edu.cn

[‡]School of Physical Science and Information Technology, Liaocheng University, Liaocheng 252059, China.

[§]School of Chemistry and Materials Science, Nanjing University of Information Science & Technology, Nanjing, 210044, China.

[±]Shaanxi Institute of Flexible Electronics (SIFE), Northwestern Polytechnical University (NPU), Xi'an 710072, China

KEYWORDS**:** thermal responsive, H_2O_2 self-sufficient, phase change materials, Domino effect, chemodynamic therapy

Materials. Iron chloride tetrahydrate (99%+) was purchased from Acros Organics. Calcium Chloride (99%), Potassium Iodide (99%+), 1,10-Phenanthroline (99%), l-α-Lecithin (98%), Gallic Acid (98%+) and Oleic acid (90%+) were purchased from Adamas-beta. Ammonia solution (25~28%) and Polyethylene glycol 200 were purchased from Shanghai Lingfeng Chemical Reagent Co.ltd. DSPE-mPEG-MW2000 (90%+) was purchased from Shanghai Zzbio Co.ltd. 1-hexadecanol (99%) was purchased from Sigma-Aldrich. Ployvinylpyrrolidone-MW8000 (K16-18) and hydrogen peroxide solution (30wt% in water) was purchased from Aladdin.

Instruments. Scanning electron microscope (SEM, S-4800, Hitachi, Japan) was applied to measure the sample morphology. Absorption spectrum was measured by UV-vis spectrometer (UV-3600 Shimadzu, Japan). The size distribution of the nanoparticles was characterized by Zetasizer Nanoseries (Nano ZS90, Malvern Instrument Ltd.). Ion concentration was determined by inductively coupled plasma mass spectrometry (ICP-MS NeXion 300X). Thermal images were obtained from an infrared camera (FLIR, Arlington, VA).

Cell Lines. Human cervical carcinoma cells (HeLa cells) were cultured under standard conditions (DMEM medium containing 10% fetal bovine serum (FBS), 37 °C and 5% CO_2).

Animal and Tumor Model. Female nude mice (5-6 weeks old) were purchased from the Comparative Medicine Centre of Yangzhou University. All animal experiments were performed according to the NIH guidelines for the care and use of laboratory animals. All the experiments were approved by the School of Pharmaceutical Science in Nanjing Tech University. The mice were subcutaneously inoculated with HeLa cells in the rear back to establish local tumor model.

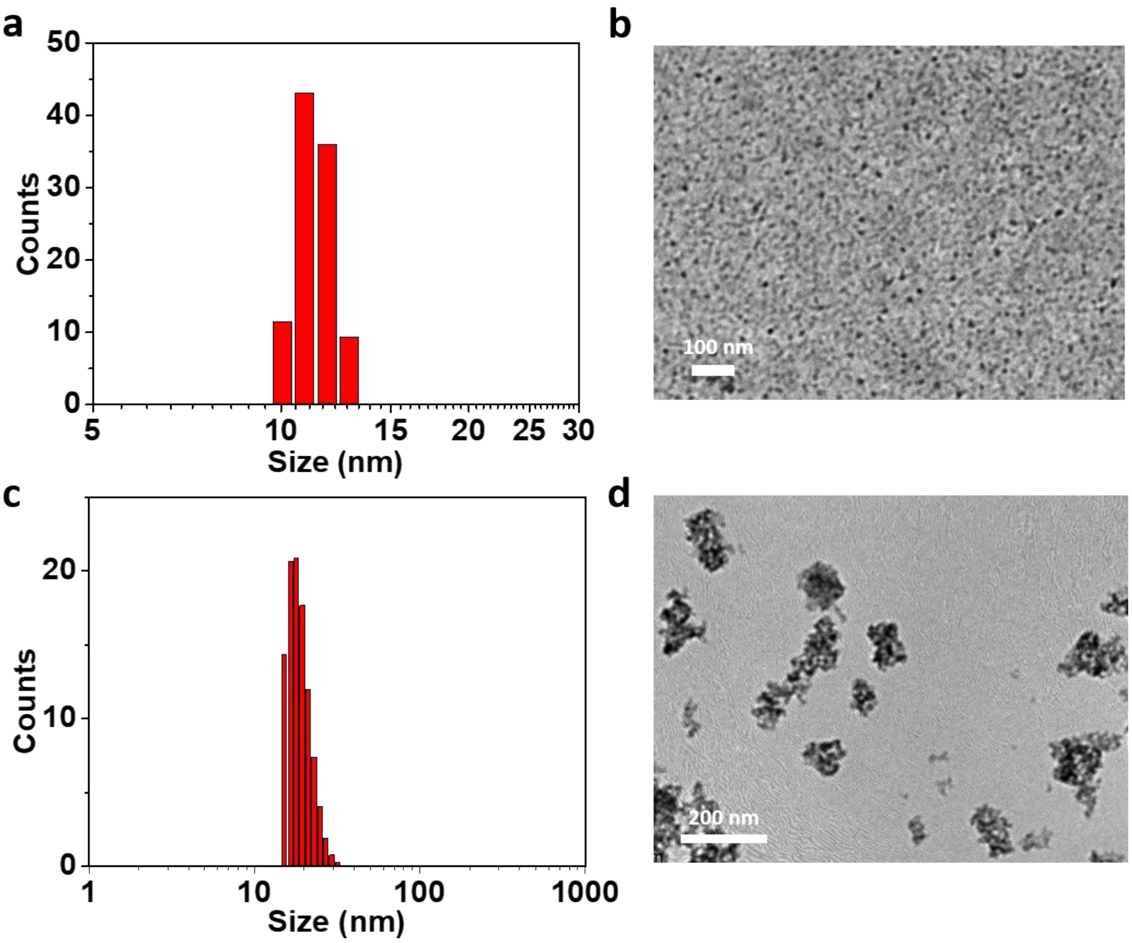

Figure S1. (a) Size distribution and (b) TEM image of Fe-GA NPs. (c) Size distribution and (d) TEM image of CaO_2 NPs.

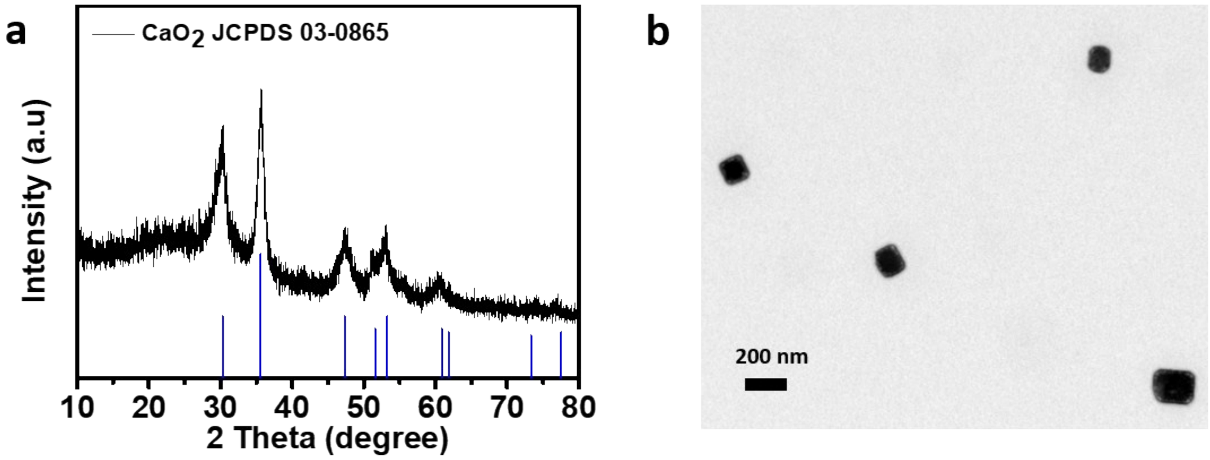

Figure S2. (a) XRD pattern of CaO_2. (b) TEM image of Fe-GA/CaO_2@PCM.

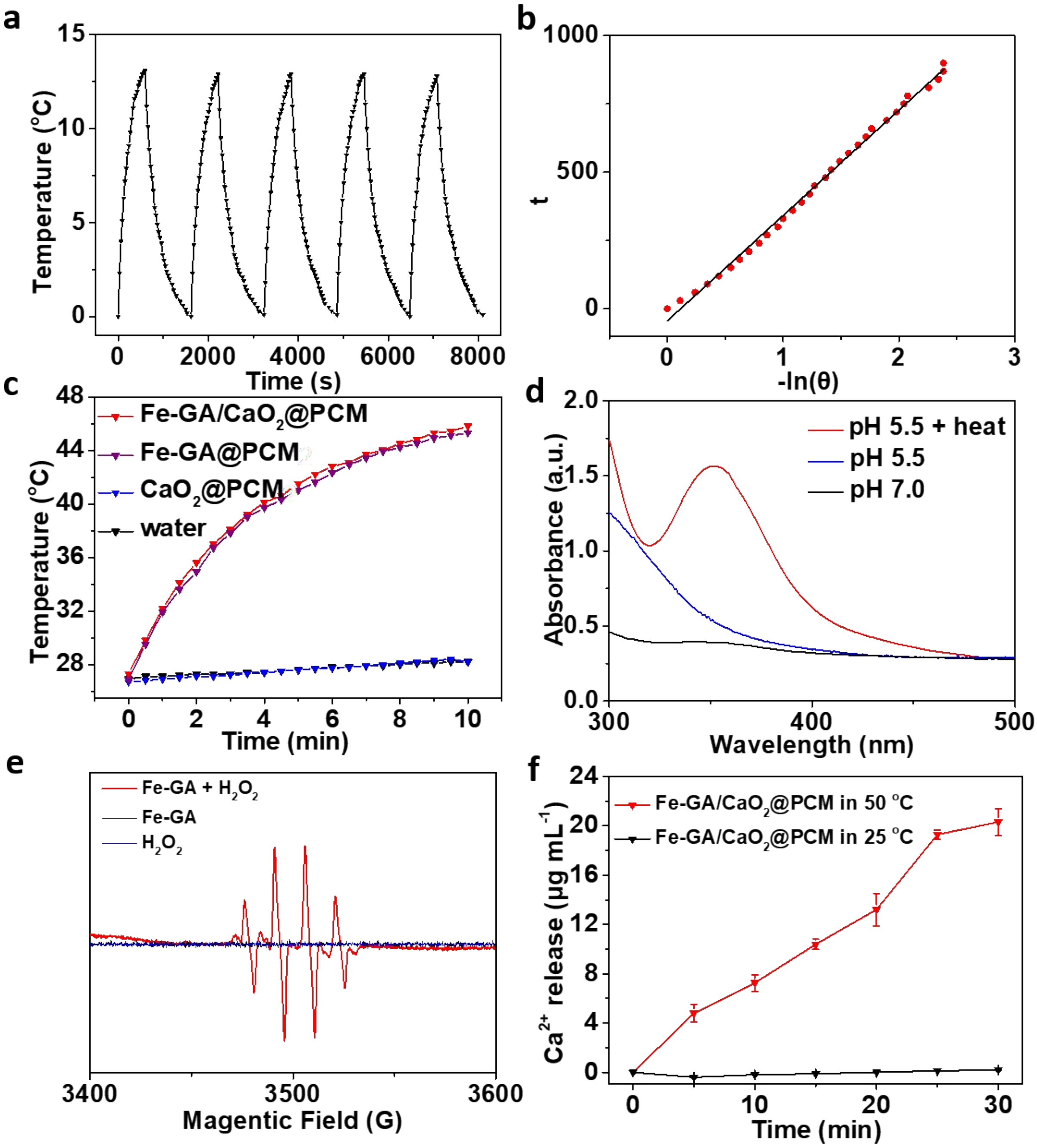

Figure S3. (a) Photothermal cycle curves of Fe-GA/CaO_2@PCM in water (808 nm laser, 1.0 W cm^{-2}). (b) Linear time data from cooling period versus negative natural logarithm of driving force temperature. (c) Photothermal heating curve of Fe-GA/CaO_2@PCM, Fe-GA@PCM and CaO_2@PCM (concentration 50 μg mL^{-1}). (d) UV-vis spectroscopy of Fe-GA/CaO_2@PCM adding with KI for H_2O_2 detection under different conditions. (e) EPR spectra to demonstrate •OH generation. (f) *In vitro* thermal response release of Ca^{2+}.

Calculation of Photothermal Conversion Efficiency

The Photothermal conversion efficiency (η) was calculated by following equations:

$$\eta = \frac{hS(T_{Max} - T_{sur}) - Q_{Dis}}{I(1 - 10^{-A_{808}})} \quad (1)$$

Where, η refers to the photothermal conversion efficiency**.** S is the surface area of cell and h is heat transfer coefficient. T_{Max} is the equilibrium temperature and T_{Sur} is the ambient temperature. Q_{Dis} is the baseline energy generated by quartz cell and water upon laser irradiation. I is incident laser power. A_{808} is the absorbance of substance at 808 nm. hS is calculated from substituting equations:

$$hS = \frac{mc}{\tau} \quad (2)$$

Where, m and c are the mass and capacity of pure water. τ is the time constant which can be calculated by following equations:

$$t = -\tau ln\theta \quad (3)$$

$$\theta = \frac{T - T_{Sur}}{T_{Max} - T_{Sur}} \quad (4)$$

Where, θ is the driving force temperature of solution**.**

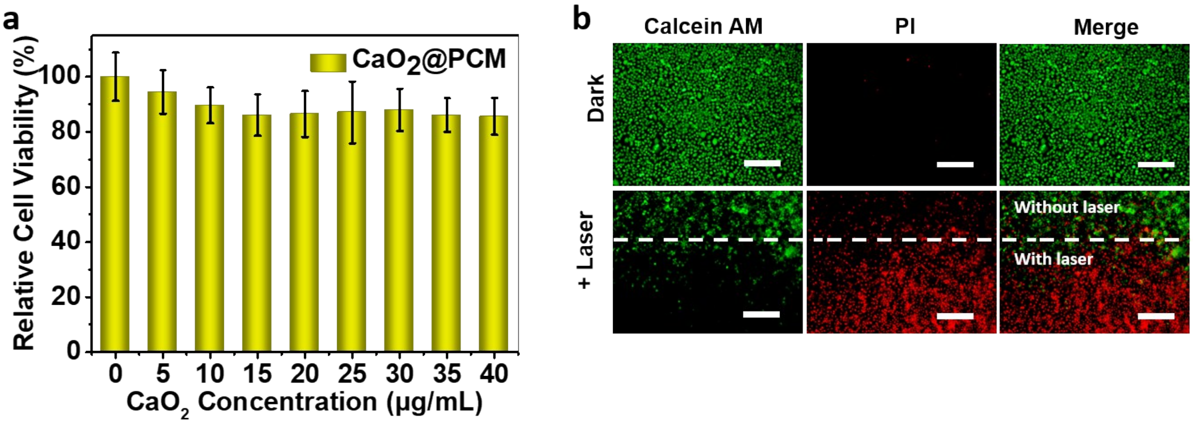

Figure S4. (a) Dark cytotoxicity assay of CaO_2@PCM NPs on HeLa cells. (b) Propidium iodide (PI) and calcein-AM double staining of HeLa cells incubated with Fe-GA/CaO_2@PCM with or without laser irradiation. Scale bars: 100 μm.

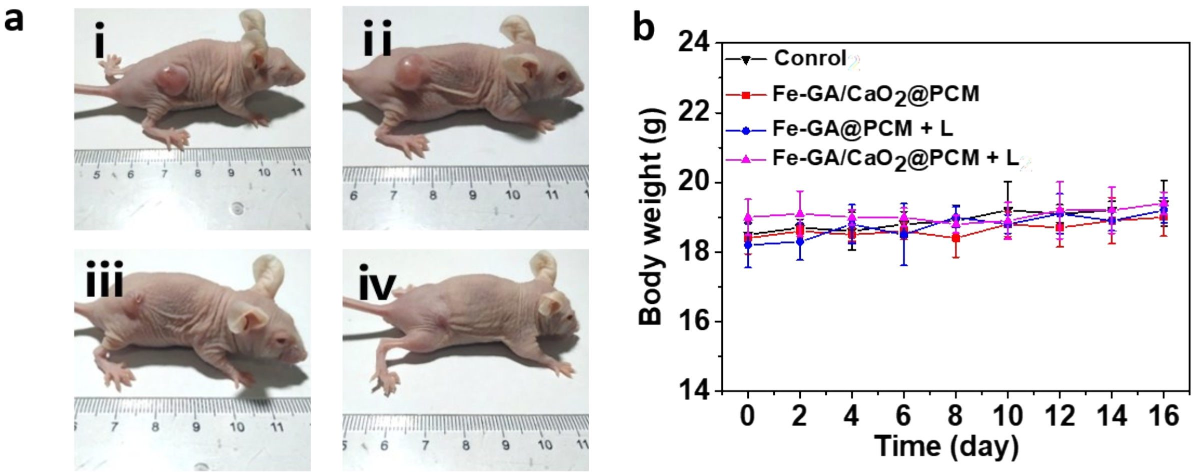

Figure S5. (a) Representative images of mice bearing HeLa tumors after various treatments. i. Saline. ii. Fe-GA/CaO_2@PCM without laser irradiation. iii. Fe-GA@PCM NPs with 808 nm laser irradiation. iv. Fe-GA/CaO_2@PCM with 808 nm laser irradiation. (b) Body weight of mice of each group during treatment.

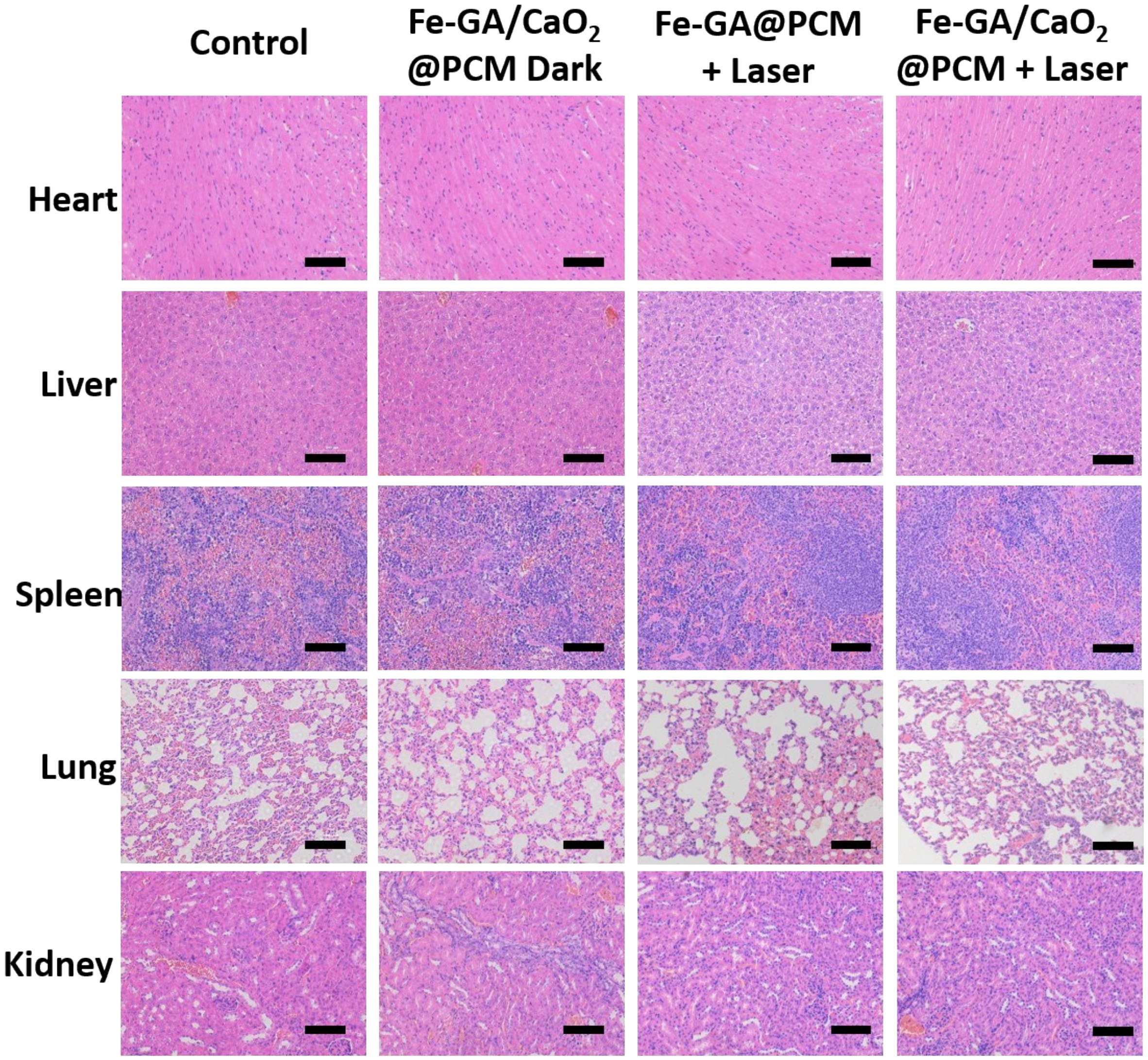

Figure S6. H&E staining of organs from each group of mice after different treatment. Scale bar: 100 μm.
